# Predicting Mitochondrial Dynamic Behavior in Genetically Defined Neurodegenerative Diseases

**DOI:** 10.3390/cells11061049

**Published:** 2022-03-19

**Authors:** Gerald W. Dorn, Xiawei Dang

**Affiliations:** Center for Pharmacogenomics, Department of Internal Medicine, Washington University School of Medicine, 660 S. Euclid Ave., St. Louis, MO 63110, USA; x.dang@wustl.edu

**Keywords:** mitochondrial dynamics, neurodegenerative diseases, mitofusin

## Abstract

Mitochondrial dynamics encompass mitochondrial fusion, fission, and movement. Mitochondrial fission and fusion are seemingly ubiquitous, whereas mitochondrial movement is especially important for organelle transport through neuronal axons. Here, we review the roles of different mitochondrial dynamic processes in mitochondrial quantity and quality control, emphasizing their impact on the neurological system in Charcot–Marie–Tooth disease type 2A, amyotrophic lateral sclerosis, Friedrich’s ataxia, dominant optic atrophy, and Alzheimer’s, Huntington’s, and Parkinson’s diseases. In addition to mechanisms and concepts, we explore in detail different technical approaches for measuring mitochondrial dynamic dysfunction in vitro, describe how results from tissue culture studies may be applied to a better understanding of mitochondrial dysdynamism in human neurodegenerative diseases, and suggest how this experimental platform can be used to evaluate candidate therapeutics in different diseases or in individual patients sharing the same clinical diagnosis.

## 1. Introduction

Aerobic life depends upon mitochondrial oxidative phosphorylation to generate ATP that fuels most biological processes. The symbiotic relationship between host cells and mitochondria, whose bacterial ancestors invaded primitive unicellular organisms approximately 1.5 billion years ago [1,2], was central to evolution of multicellular life on Earth. This relationship is inextricable, as host organisms cannot survive without their resident mitochondria, and mitochondria have exported ~99% of their DNA to host nuclear genomes. Orchestrating contextually appropriate behavior essential for metabolic homeostasis, mitochondrial biogenesis, and programmed replacement of senescent mitochondria (mitophagy) or cells (apoptosis) requires that mitochondria and host cells communicate while maintaining autonomy of individual organelles that is a legacy of their bacterial progenitors. Mitochondrial dynamism is a key component of this cell–organelle coordination [3].

Mitochondrial dynamics include mitochondrial fission, fusion, and motility [4,5]. At the observational level, these are distinct processes whose activity is determined by cell type and pathophysiological context. Thus, mitochondria in quiescent fibroblasts are part of highly interconnected networks wherein mitochondrial fusion and fission mediate continuous structural remodeling of the collective; directed subcellular transport of individual mitochondria is infrequent (~2%–4%). However, prior to fibroblast mitosis and cytokinesis, the mitochondrial network undergoes fission-mediated fragmentation, enabling around half of the resulting individual organelles to be directed into each of the daughter cell precursors [6]. By contrast, mitochondria of cardiac and skeletal myocytes are seemingly static groups of individual organelles stationed between myofilaments; fusion and fission are infrequent [7,8]. In neurons, mitochondrial dynamism proceeds according to subcellular location: mitochondria in neuronal soma exist as stationary perinuclear clusters, mitochondria within neuronal processes are either anchored in small clusters (~70%) or are actively undergoing antegrade or retrograde transport (~30%), and mitochondria within synaptic neuromuscular junctions tend to be stationary individual organelles [9,10]. The diversity of mitochondrial structure and differential application of mitochondrial dynamics according to cell type and subcellular location may explain why mitochondrial dynamic dysfunction most severely impacts the neurological system [11]. Here, we provide an overview of mitochondrial dynamism and dynamic dysfunction as they relate to genetic neurodegenerative diseases and describe an approach to their evaluation in preclinical in vitro and in vivo experimental systems.

## 2. Mitochondrial Fusion, Fission, and Motility

Mitochondrial fission, fusion, and motility are readily observed individual tangible processes. The molecular mechanisms and critical factors mediating mitochondrial dynamism are understood in great detail and were the subject of many detailed reviews [4,5,12,13]. Importantly, mitochondrial fission, fusion, and transport are physiologically and mechanistically inter-related and subject to coordinated control.

**Figure 1:**
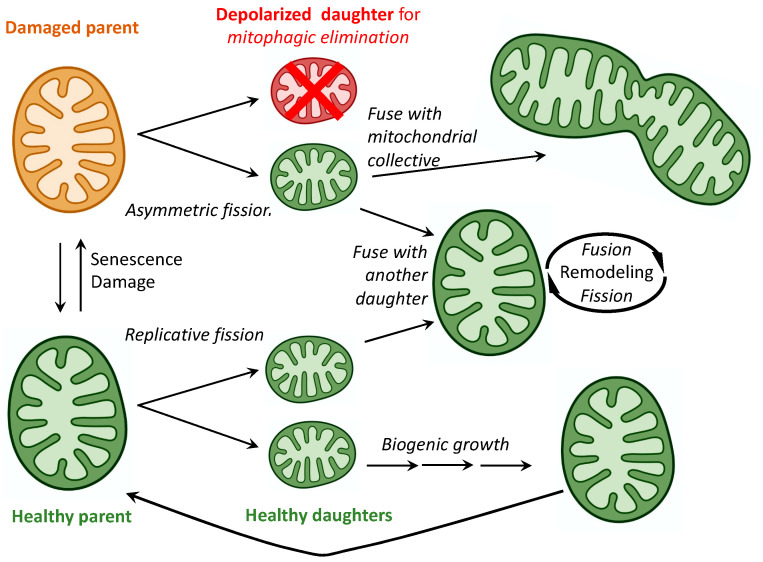
*Mitochondrial fission and fusion as determinants of mitochondrial fate*.

*Fission* of healthy mitochondria is a means for organelle replication, recapitulating ancestral bacterial reproduction [14]. To numerically increase cell mitochondria, healthy parent organelles duplicate their mitochondrial genomes and undergo *symmetrical replicative fission* into two daughter mitochondria, each of which can become a member of the host cell mitochondrial collective by: **1**. “Growing” as an individual organelle through transcription and translation of its 13 protein-coding mitochondrial DNA (mtDNA) genes and incorporation of hundreds of nuclear-encoded mitochondrial proteins (collectively termed mitochondrial biogenesis); or **2**. Joining, by fusing with an existing interconnected mitochondrial network. Thus, replicative symmetric mitochondrial fission leads both to mitochondrial biogenesis and mitochondrial fusion (Figure 1). 

By contrast, *asymmetric fission* of an injured/senescent parent mitochondrion is the process for selective removal of damaged components through their segregation and separation into one of the daughter organelles; this daughter is ultimately removed and its constituent elements recycled [15]. After asymmetric fission, the larger healthy daughter mitochondrion can undergo either biogenic maturation or integrative fusion, just as the daughters of replicative fission. However, the smaller daughter mitochondrion containing damaged elements and typically exhibiting dissipation of ΔΨm (the inner membrane electrochemical proton gradient that drives respiratory complex function) is prevented from undergoing fusion and is instead targeted for removal via mitophagy (mitochondrial-specific autophagy). Thus, asymmetric mitochondrial fission both enables and suppresses mitochondrial fusion, biogenesis, and mitophagy, depending upon the health status of the daughter mitochondria (Figure 1). Accordingly, mitochondrial fission is essential to mitochondrial quantity control (via replicative fission and biogenesis) and to mitochondrial quality control (via asymmetric fission and mitophagy). For this reason, it might be predicted that interrupting or dysregulating mitochondrial fission would seriously impact mitochondrial homeostasis. Consistent with this notion, an extremely rare, lethal, and multisystemic metabolic derangement was linked to damaging mutations of the critical mitochondrial fission protein, DRP1 [16,17,18,19], although dominant mutations in *DNM1L* *gene* can also cause dominant optic atrophy (DOA) with a relatively mild phenotype [20].

*Mitochondrial fusion* is central to postreplicative mitochondrial maturation and integration of daughter organelles into mitochondrial networks. Mitochondrial fusion is also a reparative mechanism that maintains the fitness of individual mitochondria. This process is based on the concept of repair by cross-complementation [21] (Figure 1). For example, mitochondria contain multiple independent copies of their mtDNA genomes, which accumulate mutations over time. If the mtDNA mutation burden is very high, asymmetric fission can be employed to sequester and eliminate the offending genomes (*vide supra*). However, when the mtDNA mutations are not sufficiently damaging to trigger asymmetric fission, impaired mitochondria can fuse and exchange genomes, thereby providing undamaged mtDNA templates for mutual repair [22]. The same concept of repair by complementation (or dilution) applies to mitochondrial proteins and membranes [23]. The pivotal role played by mitochondrial fusion in organelle maintenance is epitomized in cells dually deficient in the outer mitochondrial membrane fusion proteins mitofusin (MFN) 1 and 2, which not only exhibit mitochondrial fragmentation from unopposed fission, but also mitochondrial depolarization (loss of ΔΨm) from impaired fusion-mediated repair [24,25].

**Figure 2:**
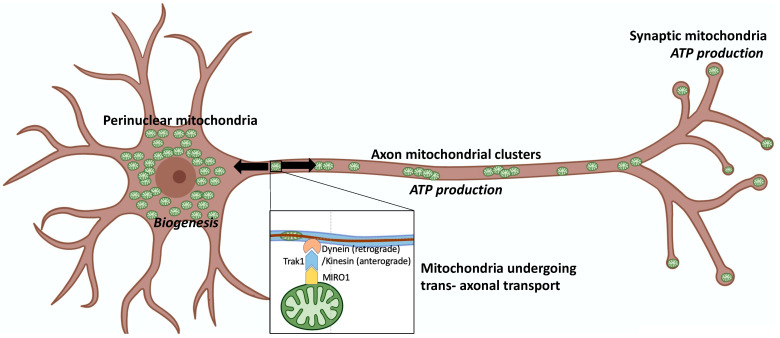
*Mitochondrial function and motility are defined by subcellular location in neurons.* Schematic depicts neuronal soma to left, axon in center, and dendrites with synapses to right. Inset shows Miro attaching a mitochondrion, via adaptor protein Trak1, to dynein or kinesin motor proteins for transport along axonal microtubules.

*Mitochondrial motility*, and especially directed transport through neuronal processes, is less completely understood than mitochondrial fission and fusion. Miro proteins on outer mitochondrial membranes interact in a calcium-dependent manner with Trak1/Milton adaptor proteins to couple mitochondria to dynein (retrograde trafficking) or kinesin-1 (antegrade trafficking) family motor proteins that transport mitochondria along cellular/axonal microtubules [26,27] (Figure 2 mitochondrial transport). 

Here, local variations in cytoplasmic free calcium, as observed in physical axonal injury, regulate mitochondrial transport and destination [27,28]. Other pathophysiological determinants that either select mitochondria for transport or direct them to particular destinations are being defined, including the Disrupted In Schizophrenia 1 (DISC1) protein that modulates antegrade kinesin-mediated mitochondrial trafficking by interacting with GTP-bound Miro1 [29] and unidentified factors related to synapse number and activity [30]. What became clear is that a pathological shift in the balance between mitochondrial fission and fusion, frequently accompanied by a disturbance in mitochondrial trafficking, is a hallmark of many clinical and experimental neurodegenerative syndromes [4,10,31,32,33,34].

## 3. Mitochondrial Dysdynamism in Genetic Neurological Diseases

Conceptually, it is easy to appreciate how mitochondrial dynamic dysfunction can manifest as neurodegenerative disease. Electrically excitable neurons have high metabolic requirements to maintain electrochemical membrane potentials and drive synaptic release and reuptake of neurotransmitters. Indeed, the neurological system has one of the highest metabolic rates of all organ systems; human brains, which comprise only ~2% of total body weight, butconsume ~20% of total oxygen (a marker for ATP production by mitochondrial respiration) [35,36]. Therefore, any genetic or environmental event that compromises mitochondrial fitness has the potential to adversely impact the neurological system.

In addition to high metabolic needs, neurons have a unique structural feature making them exceptionally vulnerable to mitochondrial dysfunction: neurons are long. Sciatic nerve motor and sensory neurons originating in the lumbar spine of a six-foot-tall person may extend ~three feet before they terminate in the feet. Mitochondrial-derived ATP is required throughout the neuron, and because ATP undergoes rapid spontaneous degradation by hydrolysis, it must be generated locally by resident mitochondria according to physiological need. The length of neuronal processes presents a formidable physical obstacle to effective mitochondrial delivery. For this reason, neurons (especially the long peripheral nerves innervating upper and lower extremities) are susceptible to damage from factors that diminish mitochondrial transport [37,38,39].

**Figure 3:**
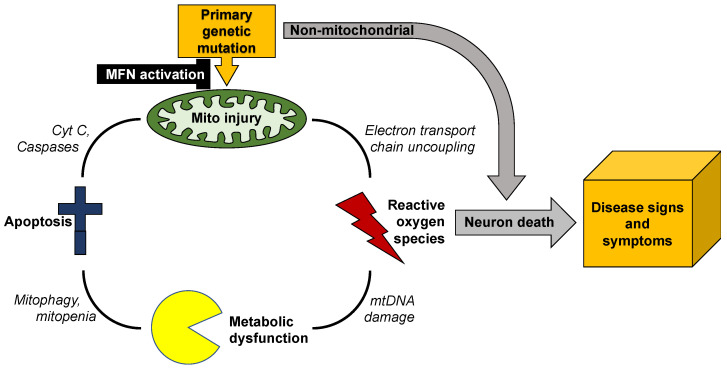
*Vicious cycle of mitochondrial degeneration in neurodegenerative diseases*. Any primary or secondary mitochondrial injury will tend to accelerate mitochondrial degeneration, culminating in neuronal death and neuromuscular dysfunction. Mitofusin (MFN) activation can provide resistance to injury through reparative fusion, potentially interrupting cycle.

Mitochondrial dysmotility is a common finding in human genetic neurodegenerative diseases, especially peripheral neuropathies (*vide infra*). The most common mitochondrial abnormality in these syndromes is mitochondrial morphological shortening, or fragmentation. In most conditions the underlying abnormality in mitochondrial dynamics is increased DRP1-mediated mitochondrial fission [40,41,42,43]. However, an impairment of MFN-mediated mitochondrial fusion is increasingly recognized as an accompanying or alternative mechanism for observed mitochondrial fragmentation [44,45,46,47]. This is an important distinction because increased mitochondrial fission does not necessarily provoke mitochondrial dysfunction, whereas suppressing mitochondrial fusion interrupts complementation-mediated mitochondrial repair and causes respiratory/metabolic dysfunction (*vide supra*) [21,22]. Moreover, mitochondrial respiratory dysfunction is damaging both because it impedes ATP production and because uncoupling of oxidative phosphorylation from ATP synthesis produces neuro- and mito-toxic reactive oxygen species (ROS). Thus, mitochondrial dysdynamism can initiate a vicious cycle of mitochondrial degeneration in which a primary impairment in the ability of individual mitochondria to undergo fusion-mediated repair evokes accelerated organelle senescence manifested in part by increased production of toxic ROS, which further injures the index organelle and can damage other members of the mitochondrial collective, which have similar dynamic impairment. Mitochondrial damage provokes more mitochondrial damage (Figure 3). 

Unless this feed-forward cycle is interrupted, mitochondrial degeneration can spread throughout the neuron, depriving it of ATP required for synaptic transmission and neuronal repair, and exposing it to ROS that will degrade nuclear DNA and damage essential proteins [48]. Because mitochondrial dysmotility and degeneration tend to be most severe distally, the ultimate consequence is neuronal die-back [49,50] or, if mitochondrial damage is more uniform, apoptotic neuron death [51,52]. This paradigm can explain why such genetically and etiologically diverse neurodegenerative diseases as amyotrophic lateral sclerosis, Huntington’s disease, and Charcot–Marie–Tooth disease share common phenotypes of mitochondrial degeneration and neuron loss.

Charcot–Marie–Tooth disease type 2A (CMT2A) is the only neurodegenerative syndrome unambiguously caused by mitochondrial dynamic dysfunction. This slowly progressive pediatric peripheral neuropathy is linked to ~100 different loss-of-function mutations of the *MFN2* gene that encodes one of the two outer mitochondrial membrane mitofusin proteins [50,53]. Clinically, autosomal dominant CMT2A typically manifests in toddlers as difficulty walking and delayed progress in ambulation; rare autosomal recessive CMT2A has a similar presentation [54]. The disease progresses during childhood and adolescence, culminating in disabling neurogenic muscular atrophy of the distal upper and lower extremities most often requiring wheelchairs for mobility and hand and foot splints to prevent secondary skeletal abnormalities [55,56]. The underlying neuropathology is progressive neuronal “die-back”, described as retrograde degeneration from the distal synapses of long peripheral motor and sensory nerves. In most instances die-back stops at approximately the knee and elbow, producing characteristic distal neurogenic muscle atrophy that spares proximal extremities. Axonal mitochondria in (directly reprogrammed) human CMT2A neurons or neurons from mice expressing human CMT2A MFN2 mutants exhibit fragmentation, depolarization (dissipated ΔΨm), and severe hypomotility [57]. Mitochondria in CMT2A neuronal soma are abnormally aggregated, forming characteristic “clumps” [58], and mitochondria can be lost from CMT2A neuronal neuromuscular junctions [57,58]. Mitochondrial fragmentation in CMT2A is the direct effect of dominant suppression of mitochondrial fusion by MFN2 mutants, while loss of inner membrane ΔΨm is likely a secondary effect of impaired fusion-mediated mitochondrial repair (*vide supra*). The exact mechanism for mitochondrial dysmotility in CMT2A is unclear, although there is evidence that MFN2 normally binds to and may therefore modulate mitochondrial Miro that couples mitochondria to the Milton/Trak transport apparatus [59]. Pharmacological activation of endogenous normal MFN1 and MFN2 (which appear functionally overlapping for mitochondrial fusion and motility), can improve mitochondrial abnormalities produced by CMT2A MFN2 mutants in neurons [25] and reverse neuromuscular degeneration in CMT2A mice [57].

Abnormalities in mitochondrial morphology and mitofusin expression/function are equally prominent in amyotrophic lateral sclerosis (ALS) [38,60] and Huntington’s disease [41,61], although the primary genetic causes of these diseases are not mutations in mitochondrial genes. ALS is an etiologically diverse upper and lower motor neuron neuropathy. Familial ALS with an identifiable genetic cause occurs in 1 out of every 10 cases [62], and mitochondrial fragmentation and impaired mitochondrial transport are typical [38,60,63,64]. By contrast, Huntington’s disease (HD) is a central nervous system cognitive and motor disorder caused by an expanded number of CAG repeats in the *huntingtin* (*HTT*) gene [65]. Mitochondrial fragmentation and depolarization are hallmarks of this condition [41], although it is not clear whether the mitochondrial abnormalities are principal contributing factors that provoke neuronal death, or reflect collateral damage that is a consequence of other primary abnormalities [66].

Mutations of the inner mitochondrial membrane fusion protein, optic atrophy 1 (OPA1), cause dominant optic atrophy (DOA) in approximately half of cases, primarily affecting retinal ganglion cells [67,68,69]. An excess of mitochondrial fission relative to fusion in retinal ganglion cell axons from OPA1 mutant mice and patient fibroblasts is evidenced by prototypical mitochondrial fragmentation [70,71,72]. Mutations of dynamin-like protein 1, which is a central effector of mitochondrial fission, are also linked to DOA. In contrast to OPA1 mutations that interrupt mitochondrial fusion, *DNM1L* mutations produce a relative excessive of mitochondrial fusion verses fission, with elongated/tubular mitochondria in patient-derived fibroblasts [20]. Thus, the balance between mitochondrial fusion and fission, rather than the absolute activity of either process, appears critical to neuronal health.

Mitochondria are not abnormal in all genetic neurodegenerative diseases, and mitochondrial dysdynamism is not necessarily a component of all neurodegenerative conditions that exhibit mitochondrial abnormalities. For example, metabolic abnormalities are described in Alzheimer’s dementia [73], a disease of uncertain and complex etiology linked to formation of extracellular deposits of β-amyloid peptide and neurofibrillary tangles of microtubular tau protein [74]. Reported mitochondrial defects, which include organelle fragmentation, are varied and inconsistent [73,75]. The sheer scope of different mitochondrial abnormalities described in Alzheimer’s disease confounds conclusions about whether they contribute in a meaningful way to, or are largely a consequence of, the primary disease. By contrast, heritable Parkinson’s disease is clearly a disease of mitochondria, since the most common genetic forms of Parkinsonism are linked to mutations of genes encoding mitochondrial PINK1 kinase (*PARK6*) that initiates mitophagy, as well as the principal mitophagy effector protein, Parkin (*PARK2*) [76]. Again, mitochondrial fragmentation is reported in some, but not all, descriptions of Parkinson’s disease [77], likely because of heterogeneity and multifactorial etiology. Finally, Friedreich’s ataxia/spinocerebellar degeneration is unambiguously a neurodegenerative disease with a mitochondrial etiology; the autosomal recessive genetic defect is in the *FXN* gene that encodes the mitochondrial iron-binding protein, frataxin. Mitochondrial iron overload in Friedreich’s ataxia compromises ATP formation by the electron transport chain and provokes excessive ROS elaboration [78,79]. It is not clear if mitochondrial dynamic dysfunction contributes to, or even exists, in this disease [80]. Variable mitochondrial phenotypes in neurodegenerative diseases and uncertainty regarding the pathophysiological impact of abnormal mitochondrial dynamics emphasize the need for analytical platforms directly relevant to individual human patients.

## 4. Evaluating Mitochondrial Dynamics

The most readily obtained and obvious evidence for mitochondrial dysdynamism is abnormal mitochondrial morphology in static images of live or fixed cells, typically imaged with a mitochondrial specific fluorophore. The most common reported mitochondrial structural abnormality is “fragmentation” or structural shortening of the organelles, conventionally and conveniently measured as reduced aspect ratio (length/width) [81,82]. Although the “normal” mitochondrial aspect ratio depends both upon cell type and physiological status [83], measuring it is conceptually and technically straightforward. Thus, a decrease in mitochondrial aspect ratio observed in high-resolution images (reduced length/width or shortening) typically reflects a relative increase in mitochondrial fission to fusion (“fragmentation”), whereas an increase in aspect ratio (“elongation”) reflects an increase in fusion relative to fission. However, altered aspect ratio does not reveal the underlying cause of dysdynamism, i.e., whether the dynamic imbalance is the consequence of altered fission, or fusion, or both. Moreover, fragmentation of mitochondria in cells having interconnected mitochondrial networks, such as fibroblasts, can be a normal component of mitosis and apoptosis [6]. Finally, short, spherical, or “fragmented” mitochondria are the norm in striated muscle cells and neuronal axons [7,8,83].

The nature of mitochondrial fusion/fission disequilibrium is best revealed by live cell imaging, using either a single mitochondrial fluorophore (e.g., Mitotracker Red, Green, or Orange), fused cells expressing different mitochondrial-targeted fluorescent proteins (e.g., Mito-GFP and Mito-RFP), or a photo-switchable mitochondrial fluorophore (e.g., MitoDendra). When a single fluorophore is used, video-confocal microscopy can document mitochondrial fusion and fission events [82]. When different groups of cells expressing mitochondrial targeted GFP or RFP are fused with polyethylene glycol, it becomes possible to quantify mitochondria content mixing from fusion as a function of time [84]. This same approach is easier using photo-switchable mito-Dendra [85], enabling a subset of mitochondria within cells of interest to be photo-switched from green to red fluorescing, and the fate of individual mitochondria (i.e., whether they are fusing, undergoing fission, or being transported within the cell) to be assessed over time. However, the photo-switching approach requires special equipment and is limited by sample throughput.

Although it is possible to infer from serial static images of differentially labelled mitochondria that subpopulations are being transported within cells, detailed quantitative assessment of the proportion as velocity of motile mitochondria requires time-lapse video-microscopy in living cells or tissue with generation of kymographs [86]. Our laboratory found this to be feasible when applied to both in vitro and ex vivo preparations. Moreover, as described below, these techniques are readily applied to either genetically manipulated mouse neurons or patient-derived cells [25,57].

## 5. Patient-Derived Primary Fibroblasts Exhibit Disease-Related Imbalances in Mitochondrial Fission/Fusion

It was challenging to devise an optimal research platform to evaluate abnormal mitochondrial fusion, fission, and motility vis a vis their relationship to human neurodegenerative diseases. Intuitively, it seems that neurons would be the best system, but primary human diseased neurons are difficult to obtain, and iPSC-derived neurons often lose seminal characteristics of the clinical disease [87,88]. The relationships between underlying genetic abnormality and mitochondrial phenotype were most extensively defined using mouse gene knockout approaches. Germ line ablation of either Mfn1 or Mfn2 is embryonically lethal [89], but fibroblasts derived from mouse embryos revealed that impaired mitochondrial fusion caused by Mfn1 or Mfn2 deletion produces mitochondrial fragmentation through unopposed mitochondrial fission; functional impairment measured as a loss of inner membrane polarization is another consequence of defective mitochondrial content exchange [90].

When relating mitochondrial dysmorphology to underlying genetic causes, one must consider the differences between gene ablation that abrogates expression of the suspect protein and is a standard experimental approach to studying genetic loss of function, versus naturally occurring loss-of-function mutations wherein a dysfunctional protein is expressed and may exert dominant suppressive effects. Thus, heterozygous ablation of the *Mfn2* gene is well tolerated in mice [89], whereas the majority of patients with CMT2A have a single mutant MFN2 allele that provokes early progressive neurodegeneration [53,55]. This difference was attributed to dominant suppression of normal MFN1 and MFN2 functioning by the single mutant allele, amplifying its functional consequences [55,56,57]. Accordingly, transgenic expression of human CMT2A mutants in mice recapitulates some CMT2A neuronal phenotypes [57,91]. However, dominant inhibition of normal mitofusins is not the only possible explanation for differences between gene knockout and expressed loss-of-function mutant effects: homozygous *Mfn2* (or *Mfn1*) gene ablation in mice does not induce neurodegeneration; it provokes early embryonic lethality [89]. Thus, changing MFN2 gene dosage by experimental “knockout” is not the same as expressing an MFN2 missense mutation. Indeed, this may be a general biological principal as gene knockdown in *Drosophila* models is a straightforward manipulation that does not induce the same quantity or magnitude of compensatory responses as expression of genetic mutants [92].

The above observations support the use of patient-derived cells carrying authentic disease-producing mutations, rather than mouse gene knockout cells, for the investigative evaluation of disease-related mitochondrial phenotypes. However, recapitulation of prototypical mitochondrial abnormalities in patient-derived fibroblasts was inconsistent. In a companion article [93], we demonstrate how mitochondrial phenotypes can be evoked by metabolic stressing in patient dermal fibroblasts from some, but not all, genetic neurodegenerative diseases. Moreover, we present data supporting the use of patient-derived cells as platforms for individualized examination of mitochondrial-directed therapeutics.

## 6. Summary and Conclusions

**Figure 4:**
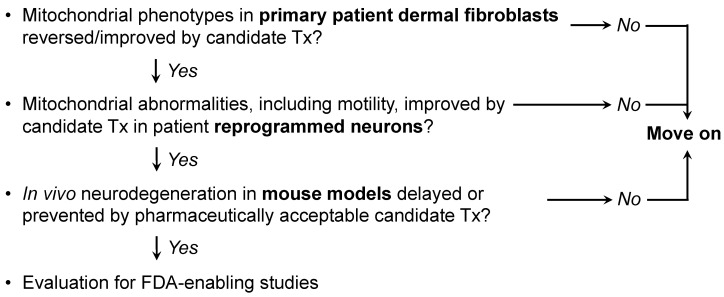
Possible screening procedure using patient-derived cells to evaluate different therapeutics for a given disease, or to assess potential efficacy of a particular approach in multiple diseases.

Mitochondrial dynamism is ubiquitous, but the relative rates and importance of mitochondrial fusion, fission, and motility differ according to cell morphology and metabolic requirements. Thus, neurons with high metabolic requirements and long axons have unique dynamic profiles and are especially susceptible to dynamic dysfunction. Mitochondrial phenotypes are difficult to evaluate in human neurodegenerative diseases because human neurons are not readily available. Previous and accompanying data demonstrate that cultured human dermal fibroblasts can be induced to exhibit neurodegenerative disease-related mitochondrial fusion-fission phenotypes by forcing mitochondrial metabolism through the simple maneuver of substituting galactose for glucose in the culture medium. Moreover, the same fibroblasts can be directly reprogrammed into neurons that retain hallmark mitochondrial abnormalities, including dysmotility, which is best measured in neuronal axons. We suggest that integration of these platforms with genetic mouse models, as depicted in Figure 4, might be an effective means of evaluating candidate therapeutics in the multitude of genetic neurodegenerative conditions exhibiting characteristic mitochondrial abnormalities, or of different patients for a personalized medicine approach.

## Data Availability

Not applicable.
